# Effects of Salinity on the Biodegradation of Polycyclic Aromatic Hydrocarbons in Oilfield Soils Emphasizing Degradation Genes and Soil Enzymes

**DOI:** 10.3389/fmicb.2021.824319

**Published:** 2022-01-11

**Authors:** Yang Li, Wenjing Li, Lei Ji, Fanyong Song, Tianyuan Li, Xiaowen Fu, Qi Li, Yingna Xing, Qiang Zhang, Jianing Wang

**Affiliations:** Shandong Provincial Key Laboratory of Applied Microbiology, Ecology Institute, Qilu University of Technology (Shandong Academy of Sciences), Jinan, China

**Keywords:** bacterial community composition, soil salinity, phenanthrene and pyrene, degradation genes, soil enzymes activity

## Abstract

The biodegradation of organic pollutants is the main pathway for the natural dissipation and anthropogenic remediation of polycyclic aromatic hydrocarbons (PAHs) in the environment. However, in the saline soils, the PAH biodegradation could be influenced by soil salts through altering the structures of microbial communities and physiological metabolism of degradation bacteria. In the worldwide, soils from oilfields are commonly threated by both soil salinity and PAH contamination, while the influence mechanism of soil salinity on PAH biodegradation were still unclear, especially the shifts of degradation genes and soil enzyme activities. In order to explain the responses of soils and bacterial communities, analysis was conducted including soil properties, structures of bacterial community, PAH degradation genes and soil enzyme activities during a biodegradation process of PAHs in oilfield soils. The results showed that, though low soil salinity (1% NaCl, *w/w*) could slightly increase PAH degradation rate, the biodegradation in high salt condition (3% NaCl, *w/w*) were restrained significantly. The higher the soil salinity, the lower the bacterial community diversity, copy number of degradation gene and soil enzyme activity, which could be the reason for reductions of degradation rates in saline soils. Analysis of bacterial community structure showed that, the additions of NaCl increase the abundance of salt-tolerant and halophilic genera, especially in high salt treatments where the halophilic genera dominant, such as *Acinetobacter* and *Halomonas*. Picrust2 and redundancy analysis (RDA) both revealed suppression of PAH degradation genes by soil salts, which meant the decrease of degradation microbes and should be the primary cause of reduction of PAH removal. The soil enzyme activities could be indicators for microorganisms when they are facing adverse environmental conditions.

## Introduction

Polycyclic aromatic hydrocarbons (PAHs) are organic molecules consisting of two or more benzene or heterocyclic rings (Patel et al., 2020), which are mainly discharged from the process of thermal decomposition and recombination of organic materials such as coal, petroleum, petroleum gas and wood in nature. Due to the recalcitrance and hydrophobicity of PAHs, a great majority are eventually deposited in soil after transformation and migration (Sun et al., 2018), leading to a serious threat to human health and ecosystem security (Tsibart and Gennadiev, 2013; Sushkova et al., 2017; Zhang et al., 2018). Among the remediation processes of PAH pollution in soils (Rivas, 2006; Ghosal et al., 2016; Kuppusamy et al., 2016, 2017; Li et al., 2020; Zhang et al., 2021), the bio-augment remediation method is considered the most suitable choice because of its low economic cost, high efficiency and sustainability (Haritash and Kaushik, 2009; Ghosal et al., 2016).

Polycyclic aromatic hydrocarbon (PAH)-degrading genes in soils are valuable biomarkers for measuring the PAH degradation potentials of bacterial communities (Wang et al., 2016). The aerobic biodegradation process of PAHs by bacteria usually dominates by dioxygenases which incorporate both atoms of oxygen molecules into the substrates (Chikere and Fenibo, 2018). Dioxygenase, a multicomponent enzyme generally consisting of reductase, ferredoxin, and terminal oxygenase subunits (Ghosal et al., 2016), is categorized into ring-hydroxylating dioxygenases (RHDs) and ring cleaving dioxygenases (RCDs) (Chikere and Fenibo, 2018). PAH-RHDα are functional genes that encode the RHD enzymes responsible for the catalysis of PAH biodegradation under aerobic conditions (Song et al., 2015). Ring-hydroxylating dioxygenase genes include the classical genes like *nah* (Habe and Omori, 2003), *phd*, *nag* (Muangchinda et al., 2015), *nid*, *pdo*, *dfn/fln*, and *nar* (Xia et al., 2015; Chikere and Fenibo, 2018). C12O, encoding catechol 1, 2-dioxygenase, associates with cleavage of the last aromatic ring in the degradation pathway of PAHs (Han et al., 2014). The quantity and expression of these genes are very important during the biodegradation of PAHs (Ghosal et al., 2016; Liao et al., 2021).

Soil enzymes are also common representations of soil biochemical characteristics, which are produced by soil microorganisms (Cortés-Lorenzo et al., 2012; Singh, 2015; Azadi and Raiesi, 2021). Soil catalase (S-CAT) can decompose hydrogen peroxide in soil and reduce the damage of excessive accumulation of hydrogen peroxide to soil microorganisms (Sun et al., 2021). Soil polyphenol oxidase (S-PPO) is an oxidoreductase that can oxidize aromatic compounds into quinones (Sullivan, 2014). Besides, soil dehydrogenase (S-DHA), reflecting the amount of active microorganisms and their degradation ability of organic matter, can be used to evaluate the degradation performance (Lu et al., 2017). The activities of these enzymes in soils are usually the most sensitive indicators to environmental changes, and their activities are always affected by soil conditions through shifting the synthesis and structure of local microorganisms (Teng and Chen, 2019; Azadi and Raiesi, 2021).

Soils in onshore oilfields are commonly suffered by multiple environmental stresses including PAHs contamination and soil salinization (Nie et al., 2009; Cheng et al., 2017). Actually, soil salts are vital factors for microorganisms during their physiological metabolic activities and important substances to maintain cells’ osmotic equilibrium (Lozupone and Knight, 2007; Rath and Rousk, 2015; Rath et al., 2019; Yang et al., 2020; Zhao et al., 2020). However, high salinity can result in dehydration or lysis of cells for microbes, then decrease microbial functions in soils (Singh, 2015; Yang et al., 2020). For microbes with salt tolerance, osmotic substances will accumulate in cells and thereby enhance the adaptation of microorganisms to salts (Hagemann, 2011; Asghar et al., 2012).

Although former studies have reported effects of the salinity on PAH degradation in soils, it is still unclear how microbial communities relate to changes of degradation genes and soil enzymes with increasing salinity. In this study, a 30-day soil remediation of PAHs under 3 salinity gradients (addition of 0, 1%, and 3% of NaCl, *w/w*) was conducted. The goals were to provide a better understanding of effect mechanisms of soil salinity on the degradation rate during a bio-augmented remediation of PAHs under salinity changes. The objectives are as follows: (1) to reveal the influence of salinity on composition and diversity of the bacterial community, and (2) to elucidate the response characteristic of functional genes and soil enzymes related to PAH degradation. The results reveal the effect extent of soil salinity on bioremediation of PAH and provide a new perspective for the assessment and remediation of PAHs in extreme environment including but not limited to oilfield soils.

## Materials and Methods

### Experimental Design

In this study, bacteria colonies were isolated and enriched directly from oil-contaminated soil in the Shengli oilfield, China. The bacteria consortium, passed on NCBI database by Yang Li (Qilu University of Technology Shandong Academy of Sciences, Jinan, China), had been proven to have a synergistic biodegradation ability for PAHs in a former experiment. The soils used in this study were collected from the Shengli Oilfield of China. The sampling site was not obviously polluted by crude oil, but had beared long-term oil exploitation since the 1960s. After air dried and ground through a 10-mesh sieve, the soils were spiked with phenanthrene (PHE) and pyrene (PYR) thoroughly to make their concentrations to 200 mg/kg and 50 mg/kg in soils, respectively. Then appropriate sterilized water was added to make the soil moisture to approximately 20%. One portion of the soil was subjected to the measurement of the basic physicochemical properties of the soil, and another was prepared for the PAH degradation experiment.

After a month of aging process, the soil was divided into three parts, named LS treatment, S1 treatment and S3 treatment, respectively. Approximately 1% sodium chloride (NaCl, *w/w*) was added to S1 treatment, and 3% NaCl (*w/w*) was added to S3 treatment. The mixture was placed in a plastic sterilized box. Each box was equipped several 0.22μm filters on the cover, in order to ensure the normal respiration of soil, and prevent the influence of microorganisms from the air. Soil samples were cultured at 25°C for 30 days. All treatments were set with 3 replicates. And during each sample collection, triplicate samples were collected for chemical and biological analysis.

### Determinations of Physico-Chemical Properties and Polycyclic Aromatic Hydrocarbons in Soils

The pH of the soil and the electrical conductivity (EC) method were used to evaluate soil salinity (Bañón et al., 2021), The percentage of weight loss of organic matter on ignition (W_*SOI*_%) method was used to determine the soil organic matter (OM) content (Nakhli et al., 2019). The obtained samples were air-dried in the shade and passed through a 60-mesh standard sieve before analysis. Ultrasonic solvent extraction technology was used to extract PAHs from soil (Pan et al., 2013; Liao et al., 2021). A high-performance liquid chromatography (HPLC) system (Agilent, United States) equipped with a fluorescence detector (RF-10AXL) was utilized to analyze PAH concentrations (Geng et al., 2022). The soil enzymes activities of S-CAT, S-PPO and S-DHA were determined as follows: enzymes were extracted from prepared soil samples by enzyme kits and the activities were determined via a microplate reader (iMark, BIO-RAD, United States) (Li et al., 2019a).

### Analysis of Microbial Community and Degradation Genes

Genomic DNA was extracted from the fresh soil samples using the Mag-Bind^®^ Soil DNA Kit M5635-02 (Omega Bio-Tek, United States). A Nanodrop 2000 spectrophotometer (Thermo, United States) was used to check the quality and concentration of the extracted DNA. The two genes (C12O and PAH-RHDα) were amplified in a triplicate and quantified using an MA-6000 real-time fluorescence quantitative PCR instrument. The primers were synthesized following former studies (Muangchinda et al., 2015; Wang et al., 2020). The reaction system was an 8 μl template dilution sample and 8 μl mixture A. The thermal cycle reaction procedure of qPCR was as follows: 5min at 95°C for stage 1, 15 s at 95°C and 30 s at 60°C for stage 2. The whole process was conducted for 40 cycles.

Shanghai Personal Biotechnology Co., Ltd was commissioned to accomplish the composition spectrum analysis of microbial community diversity. In brief, the V3–V4 region of the bacterial 16S rRNA genes was amplified with the forward primer 338F (5′-ACTCCTACGGGAGGCAGCA-3′) and the reverse primer 806R (5′-GGACTACHVGGGTWTCTAAT-3′) (Xu et al., 2021). Agencourt AMPure Beads (Beckman Coulter, Indianapolis, IN) were used for the purification of PCR amplicons, and the PicoGreen dsDNA Assay Kit (Invitrogen, Carlsbad, CA, United States) was used for quantitative measurement. After the above stages, amplicons were pooled in equal amounts, and sequencing was performed on the Illumina MiSeq platform with MiSeq Reagent Kit v3.

### Data Statistical Analysis

Before statistical analysis, Kolmogorov-Smirnov and Levene’s tests were carried out to test the normality and homogeneity of differences (Liu et al., 2021). Excel 2020 (Microsoft, United States) was used for preliminary data statistics and processing. Origin (Version 2020) (Origin Laboratories, Ltd, United States) was mainly used to draw statistical graphs. All data are derived from the mean value in triplicate. SPSS Software (International Business Machines Corp, United States) was used to analyze the differences with one-way analysis of variance (ANOVA) or a non-parametric test. Picrust2 software^1^ was used to predict the function of soil bacteria (KEGG).^2^ The community structure of bacteria was analyzed via QIIME2 and R language. To comprehensively evaluate the characteristics of microbial community diversity, alpha diversity was utilized. The Chao1 index was used to represent richness, the Shannon and Simpson indices represented diversity, and Pielou’s evenness index represented evenness.

## Results and Discussion

### The Removal Percentage of Polycyclic Aromatic Hydrocarbons in Soil

**FIGURE 1:**
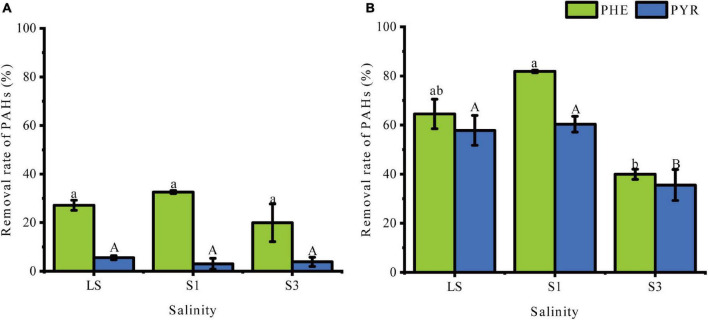
Percent removal of PHE and PYR in treatments under different salinities. **(A)** the 7^th^ day, **(B)** the 30^th^ day. Error bars represent standard deviations of triplicate samples. Different letters indicate significant differences between the different salinity treatments at *P* < 0.05.

Figure 1 demonstrates the percentage removal of PHE and PHY from soil samples at different time points. After 30 days of incubation, significant differences (*P* < 0.05) in the removal of PAHs were obtained from soils treated with different salinities. On the 7^th^ day (Figure 1A), there was no significant difference of removal rates of PHE and PYR among the three treatments (*P* > 0.05), though values of degradation rate were higher in lower salinity soils than in the higher. However, on the 30^th^ day (Figure 1B), the removal percentages of PHE and PYR in the LS treatment reached 64.52% and 57.83%, respectively, and the S1 treatment had the highest removal percentages of 81.85% and 60.33%, respectively. This indicated that appropriate salinity could probably promote the removal rate of PHE in soils (Wang et al., 2020). Compared with LS and S1, the addition of 3% NaCl (*w/w*) significantly decreased the degradation of PAHs, leading to removal of 39.95% and 35.54% for PHE and PYR, respectively. Many previous studies have revealed a similar result: decreased PAHs removal was caused by salinity stress (Ibekwe et al., 2018; Wang et al., 2019).

### Changes of Soil Properties Under Salt Stress

Soil enzymes, pH, EC and W_*SOI*_% were selected to reflect processes of biochemical reactions in the soils. As shown in Supplementary Table 1, pH values remained stable among different treatments of S1, S3 and LS and different sampling times. Soil conductivity and contents of organic matter were significantly influenced by the gradient salinities (*P* < 0.05).

**FIGURE 2:**
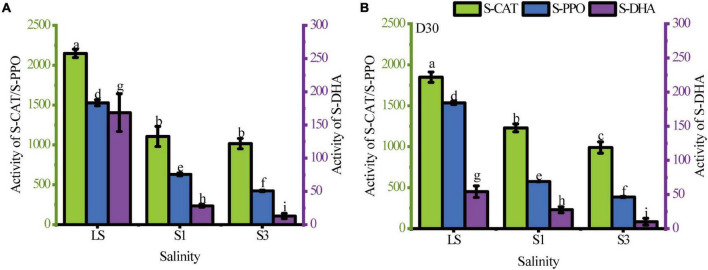
Soil enzyme activities in the LS, S1 and S3 treatments. **(A)** the 7^th^ day, **(B)** the 30^th^ day. Different letters over columns represent significant differences among treatments at the *p* < 0.05 level of LSD *post hoc* comparison tests.

Soil enzymes as catalysts of biochemical conversion and the biodegradation of PAHs have been studied intensively (Lipińska et al., 2015). In this study, the activities of three common soil enzymes (S-CAT, S-PPO and S-DHA) under different soil salinities and sampling times were analyzed to evaluate the changes in the microbial community and metabolic processes (Figure 2). Supplementary Table 2 showed the results of the difference analysis of enzyme activities between samples from the 7^th^ day and 30^th^ day. The results showed that the activities of these enzymes significantly decreased with increasing soil salinity (*P* < 0.05). All of the highest activities were found in the treatment with the lowest salinity (LS treatment).

Soil catalase (S-CAT), a common antioxidant enzyme in soil (Sun et al., 2021), can be used as an indicator of soil biomass to some extent, and soils with high biomass usually have higher catalase activity (Chabot et al., 2020). The results in Figure 1 show that the highest S-CAT activity was observed in the LS treatment, indicating that the addition of sodium chloride reduced the S-CAT activity. On the 7^th^ day, there was no significant difference in S-CAT activity between S1 and S3 treatments, but the difference became more pronounced in the two treatments as the incubation time progressed. The reduction of catalase activity in the high salt state leaded to a lower antioxidant capacity of soil microorganisms, which results in higher residual PAHs than in the low salt state.

Activities of S-PPO and S-DHA are both important enzymes for the breakdown of cyclic organic matter and represent the bioremediation capacity (Lu et al., 2017). In this study, these two enzyme activities were significantly decreased by the addition of salt (*P* < 0.05). This was the result of a significant inhibitory effect of the soil salinity on microbial degradation abilities of organic matters. Comparing the change in enzyme activity from the 7^th^ day to the 30^th^ day, the LS treatment showed the greatest change in S-DHA activity with a significant decrease of 67.89%. This may be because activity of S-DHA is an indicator of total biological activity, and bacteria without PAH-degrading abilities or that are less adapted to the environment undergo apoptosis. Li et al. (2019b) also pointed out that the increase in S-DHA activity was due to an increase in the total number of microorganisms. However, this change was absent in the treatments with relatively high salinity (S1 and S3). The reason was probably that salinity has a filter function of eliminating poorly adapted bacteria. Then the halophilic bacteria remained and were well adapted to their environment.

### Abundance of Polycyclic Aromatic Hydrocarbon-Degrading Genes in Contaminated Soil

**FIGURE 3:**
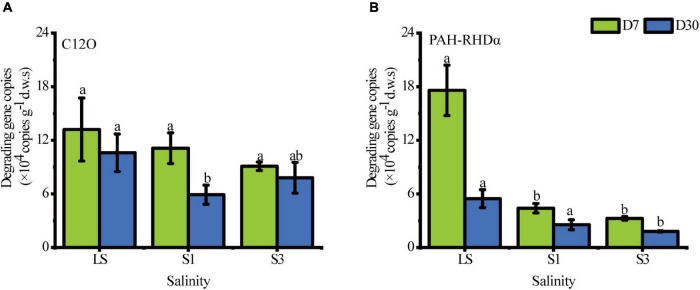
Absolute abundance of the C12O gene and PAH-RHDα gene under the different salinity treatments. **(A)** the gene of C12O, **(B)** the gene of PAH-RHDα. Different letters over columns represent significant differences among treatments at the *p* < 0.05 level of LSD *post hoc* comparison tests.

The biodegradation of PAHs in soil depends on a variety of functional genes, which are valuable biomarkers for evaluating the potential of PAH degradation (Yang et al., 2015). Real-time quantification PCR(RT-qPCR) was applied to quantify the absolute abundance of the PHA-RHDα and C12O genes (Figure 3). In general, the salt in soils gave a prominent stress to bacteria and mainly decreased the total abundance of PAH-degrading genes with salinity. The copy number of degradation genes was an indicator of PAH-degrading microbial abundance, the decrease of which signified a decrease of PAH-degrading microorganisms. All values of gene copies of PAH-RHDα in the lower salinity treatment were higher than those in higher soils. The copy numbers of the C12O gene showed an upward trend from S1 to S3 on the 30^th^ day, which meant that several PAH-degrading bacteria in the S3 treatment were halophilic and thrived under high salinity conditions. Previous studies have also reported the growth and metabolism of *Halobacillus* (Li et al., 2012), a halophilic microorganism containing the C12O gene, under high salinity (Delgado-García et al., 2018). However, there was no significant difference in the gene copies between S1 and S3 (*P* = 0.254), which could be explained by the same role played by the mildly halophilic bacteria in both the S1 and S3 treatments.

**FIGURE 4:**
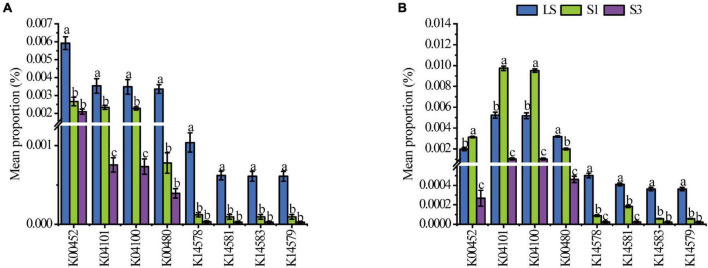
Mean proportions of the functional genes associated with the degradation of PAHs in soil after 7 days **(A)** and 30 **(B)** days of incubation. K00452 HAAO; 3-hydroxyanthranilate 3,4-dioxygenase [EC:1.13.11.6]; K04101 ligB; protocatechuate 4,5-dioxygenase, beta chain [EC:1.13.11.8]; K04100 ligA; protocatechuate 4,5-dioxygenase, alpha chain [EC:1.13.11.8]; K00480 salicylate hydroxylase [EC:1.14.13.1]; K14578 nahAb, nagAb, ndoA, nbzAb, dntAb; naphthalene 1,2-dioxygenase ferredoxin component; K14581 nahAa, nagAa, ndoR, nbzAa, dntAa; naphthalene 1,2-dioxygenase ferredoxin reductase component [EC:1.18.1.7]; K14583 nahC; 1,2-dihydroxynaphthalene dioxygenase [EC:1.13.11.56]; K14579 nahAc, ndoB, nbzAc, dntAc; naphthalene 1,2-dioxygenase subunit alpha [EC:1.14.12.12 1.14.12.23 1.14.12.24].

Picrust2 analysis was also conducted to predict the functional genes in relative quantity of each soil treatment. Eight genes associated with the PAH degradation (Li et al., 2019b) were selected to show significant variations among different treatments (Figure 4). From the 7^th^ day to the 30^th^ day, all numbers of these functional genes decreased. On the 7^th^ day, the average percentages of all genes showed the lowest values in S3 treatment and the highest in the LS. The results were associated with the bacterial genera carrying PAH degradation genes (Wang et al., 2021a). On the 30^th^ day, the average proportions of genes like k00452, k04101and k04100, increased in S1 treatment, which may be due to the abundance of bacteria containing these genes increased, and they were tolerant to the salt stress extent in the treatment of S1 (Liao et al., 2021). For the other genes, the highest abundances were only found in the LS treatment, which meant most PAH degradation bacteria were not salt-tolerant and leaded to a restrained degradation rate in high salinity soils.

### Responses of Soil Microbial Community Structure to Salt Stress

Bacteria in soils usually dominate microbial communities (Pesce et al., 2018) and play a key role in the dissipation of PAHs in soils (Li et al., 2019b). In order to discuss the effect of salt stress on the microorganisms in soils, 16S rRNA sequence was conducted to analyze the structure and diversity of bacterial communities. The results showed that salt stress caused significant differences in the formation of microbial community structure from the control treatment.

**FIGURE 5:**
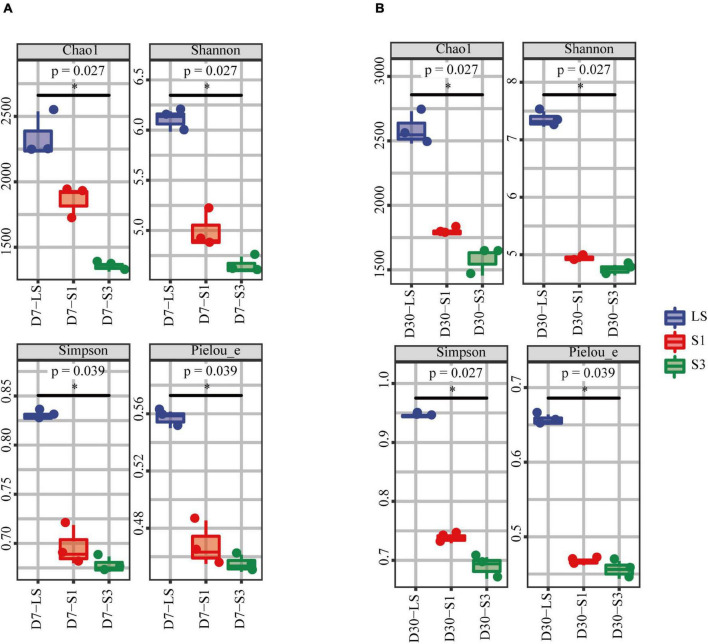
Alpha diversity index of bacterial communities in soils from the different salinity treatments. **(A)** the 7^th^ day, **(B)** the 30^th^ day.

Alpha diversity analysis was used to evaluate the bacterial diversity and richness during incubation (Liao et al., 2021). A rarefaction curve (Supplementary Figure 1) was exhibited to show the sequenced quantities of all soil samples could effectively and accurately cover and estimate all microbial communities (Xu et al., 2021). The four commonly used alpha diversity indices were shown in Figure 5, which indicates that all the mean values of alpha diversity indices followed the trend of LS > S1 > S3. That is, the higher the salinity of soils from each treatment, the lower the value of the alpha diversity index, and then the more uneven the distribution of the soil bacterial community. Considering that salinity was the only factor that varied among the treatments, the results of alpha diversity analysis further proved that salinity had an appreciable impact on soil microbial diversity, richness and evenness.

**FIGURE 6:**
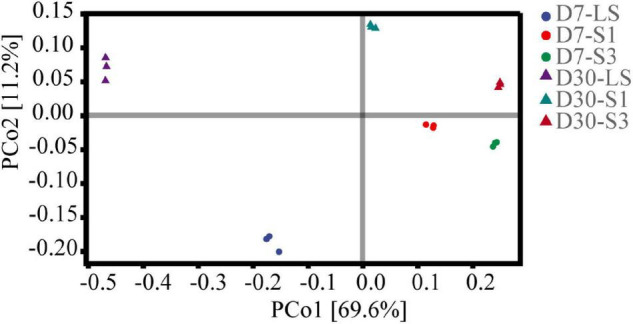
Principal coordinates analysis (PCoA) of bacterial communities in soils from the different salinity treatments.

Principal coordinates analysis (PCoA) based on Bray-Curtis distances was applied to analyze the overall structural variations of microbial structure (Figure 6). The components of PCoA1 and PCoA2 could explain 69.60% and 11.20% of the variance along their axes, respectively. The loading values of PCo1 were greatly affected by salinity and increased with the soil salinity of the treatment. In the plot, samples from different treatments separated well, which suggested significant differences among different soil salinities (*P* < 0.05). This result was consistent with the findings of alpha diversity analysis.

The statistics of taxon number under different treatments (Supplementary Figure 2) also revealed an increase in species richness over time and a decrease with salinity. The relative abundance and taxonomic analysis of soil microbial communities (Supplementary Table 3) demonstrated that *Proteobacteria* was the dominant phylum in all treatments (Cycil et al., 2020), accounting for the highest proportion of 89.20%-98.31%. Among the different treatments, the abundance was in accordance with the trend of LS < S1 < S3. The relative abundances of other phyla, including *Bacteroidetes* (0.26%–4.36%), *Firmicutes* (0.92%–3.20%), *Actinobacteria* (0.31%–3.55%), and *Chloroflexi* (0.01%–0.06%), decreased with the increase of soil salinity (De León-Lorenzana et al., 2018). *Proteobacteria, Bacteroidetes, Firmicutes, Actinobacteria*, and *Chloroflexi* have been reported to contain many genera associated with the degradation of aromatic hydrocarbons (Muangchinda et al., 2015) and to predominate in PAH-contaminated soils (Ma et al., 2016; Li et al., 2019b). The abundance of *Proteobacteria* usually increased with soil salinity (Wang et al., 2021b), and dominated the microbe communities under salt stress (Li et al., 2019a).

**FIGURE 7:**
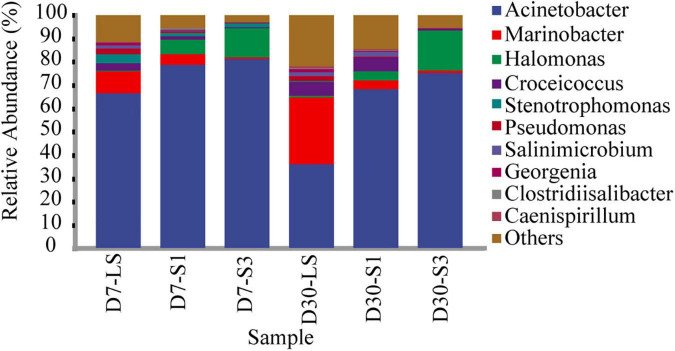
Relative abundance of different bacteria at the genus level in soils from different treatments.

Furthermore, the genus in salt-stress associated with PAH degradation deserve increasing attentions (Xu et al., 2019; Wang et al., 2020; Zhang et al., 2021). Figure 7 shows the bacterial composition at the genus level. The most frequently observed bacterial genus was *Acinetobacter*, accounting for 36.05%–81.07%, which was reported to be easier to adapt to salinity (Zhang et al., 2021). *Halomonas*, accounting for 0.28%–18.08%, showed a similar distribution characteristic to *Acinetobacter* with higher relative abundance in high salt treatment (Wang et al., 2020). In addition, the genera *Marinobacter*, *Croceicoccus*, *Stenotrophomonas*, *Pseudomonas*, and *Georgenia* were negatively affected by salinity and restrained the relative abundance. The relative abundance of other low-abundance bacteria, such as *Salinimicrobium* and *Clostridiisalibacter*, increased over time and decreased with increasing salinity (Figure 7).

**FIGURE 8:**
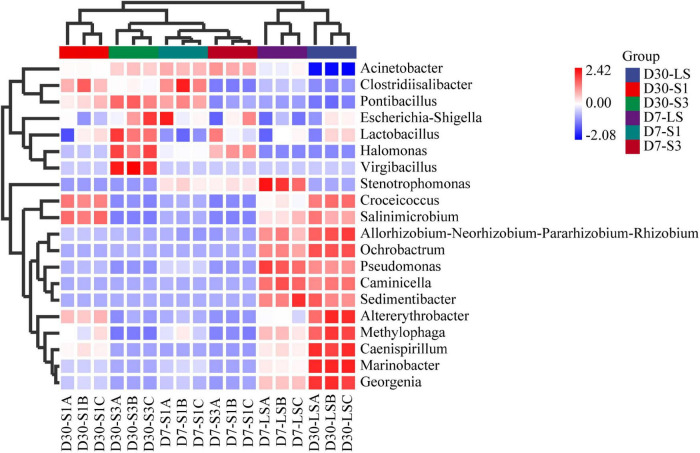
Heatmap of the top 20 genera in soils from each treatment.

Among the top 20 bacterial genera, 10 bacterial genera have been previously reported as PAH-degrading bacteria (Fernández-Luqueño et al., 2011; Kappell et al., 2014; Huang et al., 2015; Muangchinda et al., 2015; Ghosal et al., 2016; Sun et al., 2018), including *Acinetobacter*, *Marinobacter*, *Halomonas*, *Croceicoccus*, *Stenotrophomonas*, *Pseudomonas*, *Clostridiisalibacter*, *Ochrobactrum, Methylophaga*, and *Altererythrobacter*. As shown in Figure 8, the addition of salinity significantly decreased the relative abundance of some targeted genera in the treatment such as *Marinobacter*, *Salinimicrobium* etc., while others were enriched. Compared with the treatment of S1 and S3. LS treatment showed higher relative abundances of *Marinobacter*, *Salinimicrobium*, *Croceicoccus*, *Stenotrophomonas Pseudomonas*, *Orchrobactrum*, *Methylophaga*, and *Altererythrobacter* which were reported to be positively correlated with the removal percent of PAHs (Li et al., 2019b; Wang et al., 2020). *Caminicella*, *Sedimentibacter*, *Caenispirillum*, and *Gerogenia* were enriched only in the low salinity treatments, which may participate in the enhanced degradation of PAHs. In addition, salinity promoted an increase in some genera, including *Acinetobacter*, *Halomonas*, and *Clostridiisalibacter*. Moreover, the highest abundance of *Acinetobacter* and *Halomonas* appeared in the S3 treatments (Wang et al., 2020; Zhang et al., 2021). It suggested that salt application led to a decrease in soil microbial diversity, which was consistent with the results of alpha diversity. Besides, some low abundance genera associated with PAH degradation are also worth of interest and future attention, as biodegradation in complex soils occurs through synergistic interactions between bacteria (Adam et al., 2017).

### Correlation Analysis of Soil Physical and Chemical Properties, Degradation Genes, Soil Enzyme Activities and Soil Microorganisms

**FIGURE 9:**
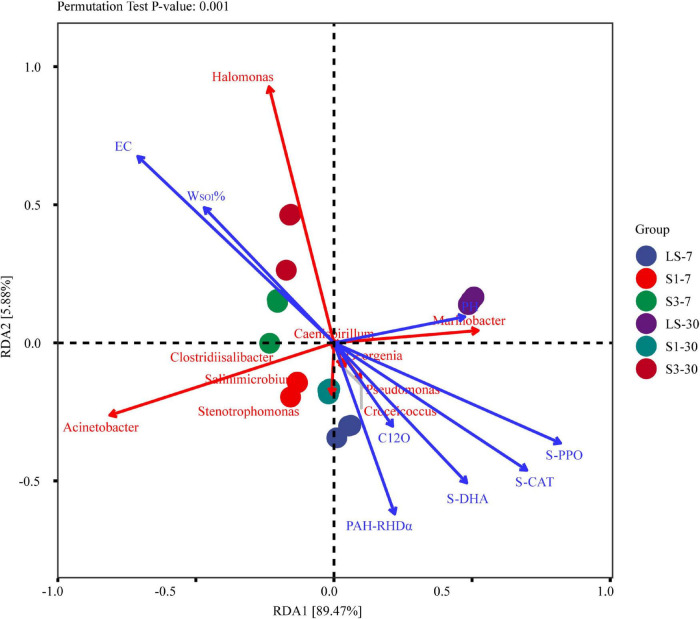
Redundancy analysis (RDA) ordination plot to show the relationships among the soil physicochemical parameters, degradation genes, enzyme activities and the relative abundance of top 10 bacterial genera. The red arrow represents the species, and the length of the arrow represents the variability of species in the sorting space. The blue arrow line represents the influencing factor, and the length represents the influence of the factor on the composition and function of the flora.

Redundancy analysis (RDA) was conducted based on the correlation between pH, EC, W_*SOI*_%, degradation genes, soil enzyme activities and the top 10 bacterial genera in relative abundance (Figure 9). The results showed that soil physico-chemical properties had a significant effect on the composition and function of the microbial community (*P* = 0.001). Electrical Conductivity value was the most important factor affecting the structure of soil flora and the relative abundance of species, followed by soil enzyme activity and organic matter content. The soil conductivities were positively correlated with the organic matter and some halophilic bacteria, such as *Halomonasas* and *Acinetobacter*, while negatively correlated with soil enzyme activities, PAH degradation, and pH. That is to say, in higher salinity treatments, the PAH degradation rate, soil enzyme and degradation genes will be lower. This is in accordance with other results discussed above in this paper.

*Halomonasas*, *Acinetobacter*, and *Marinobacter* are the three largest variants of the different species in the sorting space. The relative abundances of *Halomonas* and *Acinetobacter* were positively correlated with the soil salinity, indicating that these genera were important participants in the degradation process of PAHs during a relatively high saline environment (Czarny et al., 2020; Wright et al., 2020). However, there was a significant negative correlation between these two genera and the PAH degradation genes, indicating that these bacteria may not participate in PAH degradation directly. Wang et al. (2020) has proved that *Halomonas* cannot degrade PHE directly in experiments. The relative abundances of *Marinobacter* and other genera, including *Croceicoccus*, *Stenotrophomonas*, *Pseudomonas*, and *Salinimicrobium*, were all negatively correlated with the soil salinity, while positively correlated with pH, PAHs degradation genes and soil enzyme activities. These genera were reported to be the main force of PAH degradation in low salinity treatment (Wang et al., 2020). *Marinobacter* proved to require the cooperation of other bacteria during the biodegradation of PAHs (Cui et al., 2014), which led to a relatively low degradation rate of PAHs in high salinity soils. Soils with lower salinities had higher community diversity and richness, which led to a higher cooperation rate between different bacteria and then a higher PAH removal rate.

## Conclusion

This study illuminated the effects of salinity on the PAH removal rate, soil enzyme activities, degradation gene abundance, and the structural changes of the soil bacterial community.

(1) The PAH degradation rate increased slightly in low saline soils, while were restrained significantly in high salt conditions.
(2) With increasing of soil salinity, not only the bacterial community diversity decreased, but also abundance of degradation gene and soil enzymes. This result could be responsible for the reduction of degradation rate in saline soils.
(3) The microbial community was filtered in high salt treatments and dominated by salt-tolerant and halophilic genera, such as *Acinetobacter* and *Halomonas*.
(4) Correlation analysis confirmed that, soil salinity was negatively related with PAH degradation, abundance of functional genes and soil enzyme activities, while positively related with some halophilic genera.

## Data Availability Statement

The original contributions presented in the study are publicly available. This data can be found here: https://www.ncbi.nlm.nih.gov/bioproject/, PRJNA788045.

## Author Contributions

YL, XF, and QZ designed the study. YL, WL, and LJ performed the experiment. FS, TL, QL, and YX analyzed the data. YL, XF, and JW wrote the manuscript. All authors contributed to the article and approved the submitted version.

## Conflict of Interest

The authors declare that the research was conducted in the absence of any commercial or financial relationships that could be construed as a potential conflict of interest.

## Publisher’s Note

All claims expressed in this article are solely those of the authors and do not necessarily represent those of their affiliated organizations, or those of the publisher, the editors and the reviewers. Any product that may be evaluated in this article, or claim that may be made by its manufacturer, is not guaranteed or endorsed by the publisher.

## Footnotes

1
https://github.com/picrust/picrust2/wiki

2
https://www.kegg.jp/
