# Supplementary material for: Effects of Salinity on the Biodegradation of Polycyclic Aromatic Hydrocarbons in Oilfield Soils Emphasizing Degradation Genes and Soil Enzymes
Source: Front Microbiol. 2022 Jan 11;12:824319. doi: 10.3389/fmicb.2021.824319 (PMC8787140; doi:10.3389/fmicb.2021.824319)
Supplement: Supplementary file 1 [file Data_Sheet_1.docx]

**Supplementary material**

| **Supplementary Table 1** Main physiochemical characteristics of the soil | | | | |
| --- | --- | --- | --- | --- |
| Time(days) | salinity | **pH** | **EC(μs·cm^-1^)** | **W_SOI_%** |
| D7 | LS | 7.33±0.08 | 2882.40±458.49 | 3.63±0.03 |
|  | S1 | 7.19±0.03 | 6187.40±613.91* | 4.32±0.10* |
|  | S3 | 7.25±0.04 | 14005.85±568.87* | 4.06±0.08* |
| D30 | LS | 7.55±0.03 | 3157.10±94.47 | 3.84±0.10 |
|  | S1 | 7.52±0.03 | 6335.60±1347.89* | 5.19±0.07* |
|  | S3 | 7.40±0.01 | 14624.65±76.16* | 5.78±0.21* |
| “*” behind numbers indicate significant differences at the *P*<0.05 level in soils subjected to subjected to S1 and S3 difference from LS treatments, as determined by LSD post-hoc comparison tests. | | | | |

| **Supplementary Table 2** The difference analysis of enzyme activity between the 7^th^ day and the 30^th^ day | | | |
| --- | --- | --- | --- |
| P | S-CAT | S-PPO | S-DHA |
| LS | 0.007 | 0.825 | 0.023 |
| S1 | 0.265 | 0.013 | 0.879 |
| S3 | 0.717 | 0.009 | 0.566 |

| **Supplementary Table 3** The effect of salinity on relative abundance of top 10 bacterial phylum | | | | | | |
| --- | --- | --- | --- | --- | --- | --- |
| ID | D7-LS | D7-S1 | D7-S3 | D30-LS | D30-S1 | D30-S3 |
| Proteobacteria | 0.9379 | 0.9705 | 0.9831 | 0.892 | 0.9509 | 0.9606 |
| Firmicutes | 0.0228 | 0.0167 | 0.0093 | 0.022 | 0.0148 | 0.032 |
| Bacteroidetes | 0.0208 | 0.0061 | 0.0031 | 0.0436 | 0.0246 | 0.0026 |
| Actinobacteria | 0.0158 | 0.0052 | 0.0033 | 0.0355 | 0.0083 | 0.0031 |
| Chloroflexi | 0.0011 | 0.0006 | 0.0004 | 0.0051 | 0.0005 | 0.0006 |
| Verrucomicrobia | 0.0002 | 0.0000 | 0.0000 | 0.0003 | 0.0001 | 0.0001 |
| Patescibacteria | 0.0000 | 0.0001 | 0.0002 | 0.0001 | 0.0002 | 0.0000 |
| Acidobacteria | 0.0001 | 0.0001 | 0.0000 | 0.0001 | 0.0000 | 0.0000 |
| Cyanobacteria | 0.0001 | 0.0001 | 0.0001 | 0.0000 | 0.0000 | 0.0001 |
| Gemmatimonadetes | 0.0000 | 0.0001 | 0.0001 | 0.0001 | 0.0001 | 0.0000 |
| Others | 0.0011 | 0.0006 | 0.0003 | 0.0012 | 0.0005 | 0.0009 |

**
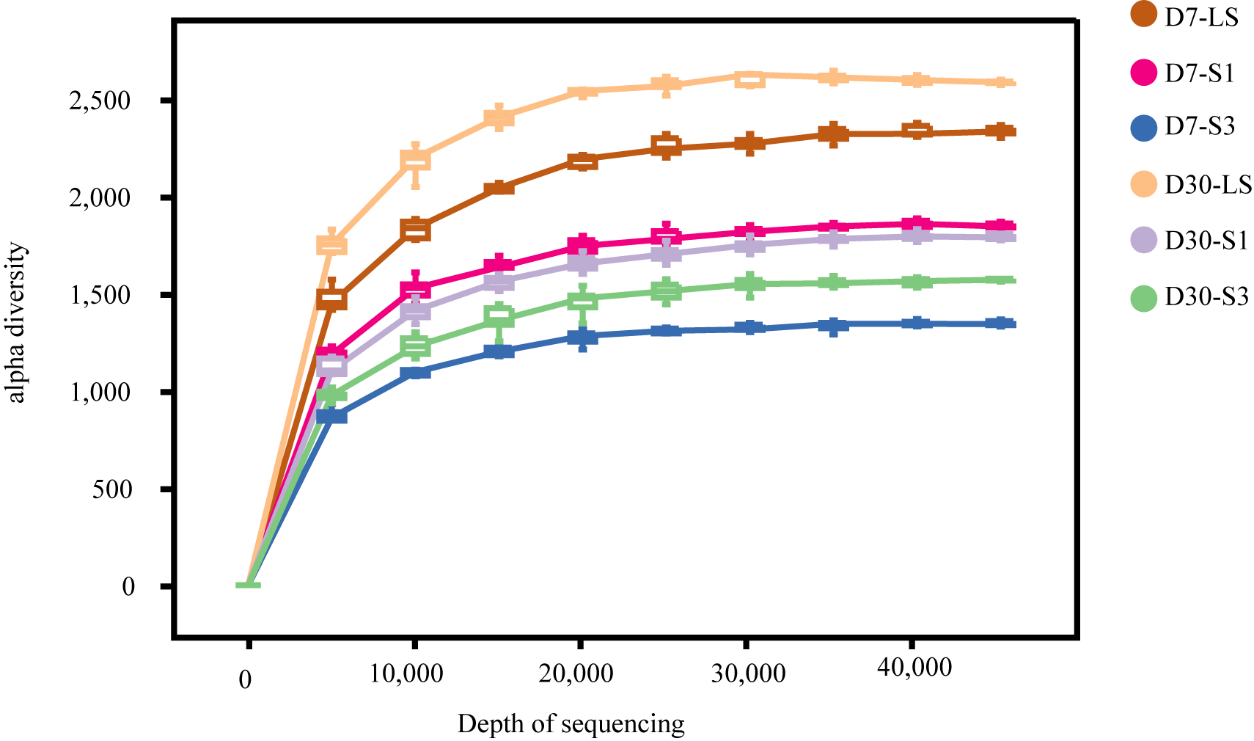
**

**Supplementary Figure 1** The rarefaction curve of different treatments

**
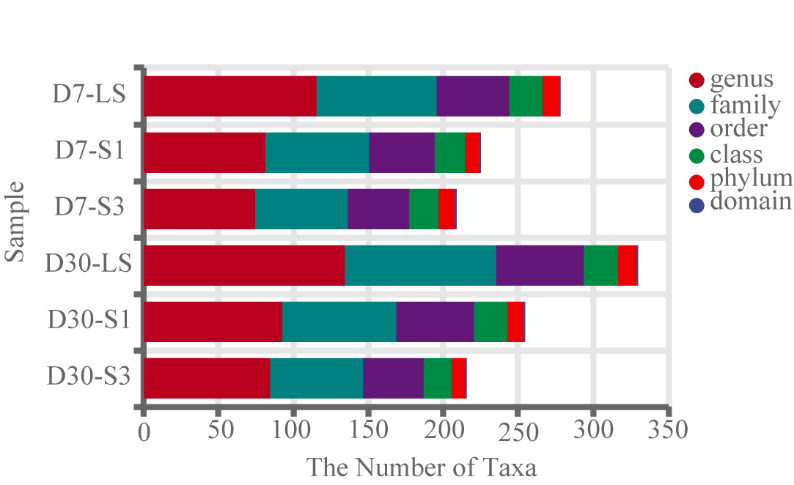
**

**Supplementary Figure 2** Statistics of taxon number under different treatments
